# Screening the optimal rTSMS frequency to orchestrate immune-fibrotic remodeling for adult spinal cord repair

**DOI:** 10.3389/fnins.2026.1845752

**Published:** 2026-06-16

**Authors:** Xiaotong Hou, Siyuan Zhou, Qianfeng Wang, Hengfei Sun, Hongbo Yu

**Affiliations:** 1 State Key Laboratory of Brain Function and Disorders, School of Life Sciences, Fudan University, Shanghai, China; 2 Institute of Science and Technology for Brain-Inspired Intelligence, Fudan University, Shanghai, China

**Keywords:** fibrotic scar reduction, frequency-specific, label-free imaging, M2 microglia, repetitive trans-spinal magnetic stimulation, spinal cord injury

## Abstract

**Introduction:**

The clinical translation of magnetic stimulation for central nervous system trauma is severely hindered by “parameter ambiguity”—the lack of evidence-based screen of stimulation protocol. Repetitive trans-spinal magnetic stimulation (rTSMS) holds therapeutic promise, yet its frequency-dependent effects on the complex spinal microenvironment remain poorly understood. To address this gap, this study aimed to decode the frequency-response relationship of rTSMS and establish an optimal, evidence-based frequency standard to orchestrate immune-fibrotic remodeling and promote functional recovery following spinal cord injury (SCI).

**Methods:**

Utilizing novel *in vivo* label-free second-harmonic generation (SHG) imaging to visualize the real-time microglia activation, we performed a high-fidelity close-loop screen of various rTSMS frequencies (10 Hz, 40 Hz, and 80 Hz). In an adult mice SCI model, we integrated transcriptomic profiling, morphological analysis, electrophysiological recordings, and behavioral assessments to comprehensively evaluate the neuroregenerative potential.

**Results:**

We identified 40 Hz as a privileged therapeutic frequency that specifically modulates microglia and the extracellular matrix. Unlike 10 Hz or 80 Hz regimens, 40 Hz rTSMS uniquely reprogrammed the transcriptomic landscape, driving microglia toward a restorative M2 phenotype, and most importantly, suppressing collagen fibrillogenesis. This targeted modulation effectively attenuated fibrotic scarring and preserved mitochondrial dynamics and axonal integrity. Consequently, the 40 Hz protocol significantly enhanced corticospinal tract conduction and drove robust, long term sensorimotor recovery.

**Conclusion:**

Our findings define 40 Hz as the critical therapeutic standard for coupling immune modulation with fibrotic remodeling in SCI. By overcoming the barrier of inconsistent parameters, this study provides a precise, clinically translatable framework for the application of rTSMS in neurorestorative medicine.

## Introduction

Spinal cord injury (SCI) represents a devastating central nervous system (CNS) disorder (Satkunendrarajah et al., 2018; Shibahashi et al., 2019). Over the past few decades, the global prevalence of SCI has steadily risen (Khorasanizadeh et al., 2019). However, the intricate complexity of SCI mechanisms means that a curative solution remains elusive, imposing a substantial burden on families and society (Mofidi et al., 2025).

The pathophysiology of SCI is notoriously complex. The initial primary mechanical trauma triggers a cascade of destructive secondary injuries, involving ischemia, mitochondrial dysfunction, oxidative stress, inflammatory responses, and apoptosis (Yang et al., 2005; Donnelly and Popovich, 2008; Ahuja et al., 2017; Hu et al., 2023; Zuo et al., 2023). This secondary injury phase typically persists for two weeks and is considered the critical therapeutic window. Subsequently, the pathology progresses to a chronic phase characterized by the formation of a dense fibrotic scar at the lesion epicenter surrounding by glial scar (Aimone et al., 2004; Hu et al., 2010; Göritz et al., 2011; Adams and Gallo, 2018; Nemati et al., 2025). Coupled with the extremely limited regenerative capacity of the adult mammalian CNS, regenerating synapses are unable to traverse the lesion, resulting in long-term, irreversible functional impairment (Tran et al., 2018; Dias et al., 2018; Katoh et al., 2019). Despite decades of research, effective clinical treatments capable of reversing this damage have yet to be realized (Courtine and Sofroniew, 2019). Therefore, there is an urgent need to establish safe, effective, and translatable therapeutic strategies to promote spinal cord repair.

Microglia, the primary resident immune cells of the CNS, play pivotal roles in development, homeostasis, and neurological disease (Hanisch and Kettenmann, 2007; Colonna and Butovsky, 2017; Li and Barres, 2018; Prinz et al., 2019; McNamara et al., 2023). Following SCI, microglia are among the first responders; they immediately detect microenvironmental changes and rapidly migrate to the injury site (David and Kroner, 2011; Orr and Gensel, 2018; Brennan et al., 2022). It is generally accepted that activated microglia can be broadly categorized into two distinct phenotypes: the pro-inflammatory M1 and the anti-inflammatory M2. Dysregulation of the M1/M2 ratio has long been identified as a key factor in regeneration failure (Kigerl et al., 2009; Hu et al., 2015; Gensel and Zhang, 2015; Tang and Le, 2016). M1 microglia release cytotoxic factors such as TNF-*α* and IL-1β, exacerbating secondary injury and killing surrounding healthy neurons. Conversely, M2 microglia secrete anti-inflammatory and neurotrophic factors that promote axonal regeneration and protect surviving neurons (Tang and Le, 2016; Cherry et al., 2014). Furthermore, they are also central to the formation and remodeling of the injury scar, interacting closely with astrocytes to form the glial scar and modulating the deposition of collagen by fibroblasts in the fibrotic scar (Bellver-Landete et al., 2019; Yu et al., 2024). Notably, Li et al. revealed that neonatal mice achieve “scar-free” healing with complete axonal regeneration—a phenomenon orchestrated specifically by microglia (Li et al., 2020). Yet, in the pathological context of adult mammals SCI, the failure to effectively phagocytose and remodel the dense collagenous and glial barriers physically impedes regeneration (Tran et al., 2018; Dias et al., 2018; Katoh et al., 2019; Kopper and Gensel, 2018). Thus, recruiting microglia to synergistically resolve inflammation and remodel the scar barrier represents a promising and underutilized avenue.

Transcranial Magnetic Stimulation (TMS), a non-invasive, painless, and relatively safe neuromodulation technique, is widely used in psychiatry and rehabilitation medicine (Hallett, 2000; Kobayashi and Pascual-Leone, 2003; Polanía et al., 2018; Lee et al., 2021; Somaa et al., 2022). Emerging evidence suggests that TMS not only triggers neuronal firing but also possesses the potential to modulate neuronal function and improve the CNS microenvironment (Cho et al., 2012; Klomjai et al., 2015; Chervyakov et al., 2015; Sveva et al., 2025), offering a unique non-pharmacological intervention for neurotrauma. Although studies have indicated the potential of TMS to modulate microglia, its application remains limited by a lack of unified, evidence-based standards for stimulation parameters, particularly frequency. For instance, 10 Hz repetitive TMS (rTMS) has been shown to induce M2 microglial polarization in the rat cerebral cortex (Luo J. et al., 2022) and inhibit M1 polarization via pathways such as HMGA2/NF-κB in models of cortical neuroinflammation (Hong et al., 2022). Similarly, intermittent Theta Burst Stimulation (iTBS) has been found to suppress microglial inflammation in rodents (Zong et al., 2020; Luo L. et al., 2022; Zhu et al., 2024), with similar effects observed under 20 Hz rTMS (Caglayan et al., 2019; Qian Z. et al., 2024; Qian F. et al., 2024) and 25 Hz rTMS (Cao et al., 2022). However, conflicting evidence highlights the complexity of parameter selection. For instance, iTBS has been reported to exacerbate neuroinflammation and promote M1 polarization (Muri et al., 2020). Although 10 Hz rTMS can promote microglia-mediated neuronal plasticity, the cellular morphology and dynamic surveillance behavior of microglia remain unchanged (Eichler et al., 2023). Furthermore, certain high-frequency protocols like 100 Hz rTMS, despite effectively enhancing neuronal excitability, fail to alter microglial density (Zorzo et al., 2019). Similarly, administration of 1 Hz rTMS to rats did not alter microglial density, expression of reactive markers, or phagocytic properties (Liebetanz et al., 2003). Regarding SCI applications, most research has focused on stimulating the primary motor cortex (Michel-Flutot et al., 2022; Kesikburun et al., 2023; Benavides et al., 2025; Xu Q. et al., 2025a). Repetitive Trans-spinal Magnetic Stimulation (rTSMS), which applies stimulation directly to the spinal lesion, has been explored at various frequencies (e.g., 1 Hz, 10 Hz, 20 Hz) and is claimed to promote repair (Chalfouh et al., 2020; Zhai et al., 2024; Song et al., 2025; Wu et al., 2026), yet comprehensive studies remain scarce.

To systematically optimize rTSMS parameters, precise monitoring of cellular dynamics *in situ* is essential. Second Harmonic Generation (SHG) imaging offers a powerful, label-free optical solution for this challenge. SHG signals are derived exclusively from non-centrosymmetric structures, such as the polymerized microtubules within the cytoskeleton (Fine and Hansen, 1971; Campagnola and Loew, 2003; Dombeck et al., 2003). Our laboratory has previously demonstrated the efficacy of SHG in tracking the real-time movement and aggregation of microglia within the cerebral cortex (Sun, 2019; Sun et al., 2022). Building on this established protocol, we propose that SHG can serve as an effective readout to evaluate microglial motility following rTSMS intervention.

Despite the potential of rTSMS, a critical knowledge gap remains: there is no consensus on evidence-based standard stimulation parameters effectively recruit microglia to the injury site, nor is there clarity on the functional consequences of such recruitment. Microglia are a double-edged sword; indiscriminate activation could exacerbate damage rather than resolve it. To address these challenges, we employed SHG imaging to systematically screen rTSMS frequencies, establishing 40 Hz as the optimal parameter for maximal microglial manipulation. Furthermore, we elucidated the underlying mechanisms, demonstrating that frequency-specific rTSMS effectively polarizes microglia toward a restorative phenotype. This shift attenuates neuroinflammation and actively remodels fibrotic scarring. By deciphering these pathways, our study provides a standardized, evidence-based rTSMS framework to foster a regenerative milieu after SCI.

## Materials and methods

### Mice

All animal procedures were performed in accordance with the guidelines provided by the National Institutes of Health (NIH) and were formally approved by the Animal Care and Use Committee of Fudan University. The animal protocol was approved under approval number [SYXK-Hu-2020-0032]. Adult wild-type C57BL/6 mice were sourced from Shanghai SLAC Laboratory Animal Co, Ltd. In alignment with NIH policy on sex as a biological variable, both male and female mice were utilized across all experimental cohorts. Preliminary assessments indicated no significant sex-specific differences in functional outcomes or physiological responses; consequently, data from both sexes were pooled for subsequent analyses.

Mice were euthanized at experimental endpoints by deep isoflurane anesthesia followed by cervical dislocation. Briefly, mice were placed in an induction chamber with 5% isoflurane until loss of consciousness, then maintained at 3% isoflurane delivered via a nose cone. The depth of anesthesia was confirmed by the absence of a withdrawal reflex to toe pinch. Subsequently, cervical dislocation was performed as a physical method to ensure death, in accordance with the AVMA Guidelines for the Euthanasia of Animals.

### Surgeries

*T10 laminectomy*: mice were anesthetized with isoflurane (5% induction, 1–3% maintenance) supplemented by local lidocaine (4 mg/kg). A 1.5 cm midline incision was made at the T10 level to expose the paraspinal muscles. Following muscle retraction, the T10 lamina was thinned using a micro-drill and meticulously removed to expose the dural-enclosed spinal cord. Proper exposure was confirmed by the visualization of the intact spinal cord and the dorsal median vein, ensuring no iatrogenic injury to the neural tissue.

*Spinal cord window preparation*: following T10 laminectomy, a 3-mm-diameter glass coverslip was positioned directly over the exposed spinal cord. To stabilize the cord and eliminate motion artifacts, 2% agarose (pre-cooled to 39 °C) was applied to the margins of the coverslip, allowing it to infiltrate the sub-coverslip space via capillary action to create a sealed interface. A custom-fabricated acrylic mounting frame was then secured to the surrounding laminae and adjacent tendons using cyanoacrylate tissue adhesive (1469SB Vetbond, 3 M Co., USA), with the imaging window centered within the frame. To ensure rigid stabilization for intravital imaging, dental acrylic resin was prepared in a pre-cooled porcelain bowl and applied to fill the gap between the acrylic frame and the tissue layer. The perimeter of the glass coverslip was hermetically sealed with the resin while maintaining a clear optical aperture. The assembly was allowed to polymerize for 10 min. Notably, to maintain experimental uniformity, this standardized closure and stabilization protocol was performed at the T10 segment for all mice across all experimental cohorts, regardless of whether they were subjected to subsequent imaging.

*Spinal cord crush injury model*: following T10 laminectomy, a complete crush injury was induced by compressing the spinal cord for 2 s using 0.2 mm-wide micro-forceps. Successful modeling was confirmed by immediate intraparenchymal hemorrhage and edema at the lesion site, followed by the manifestation of complete hindlimb paralysis (BMS score = 0), tail immobility, and urinary retention upon recovery from anesthesia. Post-operatively, mice received intraperitoneal saline (0.5 mL) and thermal support until fully resuscitated, with food and hydration provided at the cage floor level. Manual bladder expression was performed twice daily for the duration of the study to ensure animal welfare and prevent secondary complications.

*Electrode implantation*: biocompatible flexible carbon nanotube (CNTs) Wang et al., 2020; Wang et al., 2024 were employed as stimulation electrodes in this study. Following the T10 laminectomy, a small opening was gently made in the dura mater using a fine needle. The CNTs were then carefully inserted into the spinal cord at an oblique angle through the dural puncture. To ensure initial stability, the electrodes were secured to the mounting backplate with cyanoacrylate adhesive. Subsequently, a glass coverslip was positioned over the laminectomy site to further immobilize the electrodes. The coverslip was adhered with tissue glue, and the entire construct was hermetically sealed and reinforced with super dental bond (Super-Bond C&B, Sun Medical, Japan) to provide rigid fixation of both the electrodes and the mounting frame.

### MCS treatment

MCS was delivered to the spinal cord through the implanted CNT interfaced with metal leads. The stimulation protocol utilized charge-balanced, symmetric biphasic current pulses to ensure electrochemical stability. Each pulse had a duration of 200 μs per phase, resulting in a total pulse width of 400 μs (STG3000, MultiChannel Systems, USA). Stimulation was administered at a constant frequency of 40 Hz for a total duration of 10 min per session. To achieve an optimal balance between therapeutic efficacy and tissue safety, a microcurrent intensity of 40 μA was strictly maintained throughout the procedure.

### rTSMS treatment

rTSMS was administered to mice in the awake state using a MagVenture magnetic stimulator (MagPro R30, MagVenture, Denmark) equipped with a figure-eight coil (C-B60, MagVenture, Denmark). During the intervention, mice were manually restrained in a customized fabric wrap, with the center of the coil positioned directly over the target spinal segment. The stimulation intensity was set at 30% of the maximum stimulator output. The standard protocol consisted of 200 cycles, with each cycle comprising a 0.5 s stimulation period followed by a 2 s inter-train interval. The protocol was based on a practical balance between achieving a total session duration close to the commonly reported 10-min benchmark (Chalfouh et al., 2020; Zhai et al., 2024; Song et al., 2025; Wu et al., 2026) and preventing coil overheating during prolonged high-frequency operation. Stimulation frequencies were adjusted according to the specific experimental cohorts (10, 40 and 80 Hz). To ensure a rigorous double-blind design, one researcher performed the SCI surgeries, while an independent investigator, blinded to the surgical procedure, was responsible for randomizing mice into the Sham or rTSMS groups and administering the daily interventions.

### SHG imaging system *in vivo*

Multimodal nonlinear optical imaging was performed using a protocol previously established for cerebral imaging (Sun et al., 2022), adapted here for the spinal cord. Briefly, imaging was conducted on a commercial laser-scanning microscopic system (Sutter Instrument, USA) integrated with a mode-locked Ti:sapphire femtosecond laser (100 fs pulse width, 80 MHz repetition rate; Spectra-Physics, USA). Excitation was set at 870 nm with linear polarization, delivering approximately 80 mW of average power to the spinal tissue. A 20 × water-immersion objective (Plan-Apochromat, NA = 0.95; Olympus, Japan) focused the excitation beam and collected backward-reflected (epi-detection) signals. Second-harmonic generation (SHG) and two-photon excited fluorescence (TPEF) signals were detected simultaneously through independent channels equipped with a 435/10 nm bandpass filter (Olympus, Japan) and a 610/75 nm bandpass filter (Chroma, USA), respectively. All images were acquired with a 16-bit pixel depth at a scanning speed of 7.78 μs per pixel.

### Image processing and quantitative analysis

Data analysis followed previously described standardization and quantification procedures. Three-dimensional (3D) Z-stacks were acquired at 1 μm intervals and aligned based on maximal correlation coefficients to generate averaged 2D maps. For longitudinal multi-day comparisons, signal intensities were calibrated against the auto-fluorescence signal from the 610 nm channel to ensure cross-session consistency. Image processing and statistical analyses were performed using MATLAB (version 9.8) and OriginPro.

### Microglia depletion

PLX3397 from Sysebio (Changzhou, China) was formulated in American Institute of Nutrition 76A (AIN-76A) standard chow (475 mg/kg). Mice were fed a PLX3397 diet for 4 weeks to eliminate microglia.

### RNA extraction and RNA-seq

After a quick PBS perfusion, spinal cords of adult mice were rapidly dissected out 15 days after the crush injury. The spinal cords were divided into different groups according to the frequency of rTSMS (non-SCI, sham, 40 Hz, 10 Hz, and 80 Hz). To satisfy the minimum sample size requirements for subsequent analysis, 1-cm spinal cord segments centered on the lesion epicenter from seven mice within the same treatment group were pooled to form a single biological replicate. Each experimental group comprised three such replicates. All collected tissues were immediately preserved in 1.5 mL of RNA-Locker (Sangon Biotech) and stored according to the manufacturer’s instructions for further molecular processing. Total RNA extraction, quality assessment, and high-throughput sequensing were performed by a commercial service provider (Sangon Biotech, Shanghai, China) following standardized protocols.

### Bioinformatics and statistical analysis

Differential gene expression analysis was performed using the DESeq2 package in R to quantify transcriptional changes between experimental conditions. For each gene, logarithmic fold change (log_2_FC) and *p*-values were calculated based on normalized count data. To adjust for multiple comparisons, *p*-values were corrected using the Benjamini–Hochberg procedure (adjusted p value).

GSEA was conducted to evaluate pathway-level biological trends using the fgsea R-package. A ranked list of all detected genes, ordered by log_2_FC (descending), was generated without prior filtering. Reference gene sets for *Mus musculus*—including GO Biological Processes and Reactome pathways—were retrieved from the Molecular Signatures Database (MSigDB) via the msigdbr R-package. The analysis was configured with 10,000 permutations, restricting gene set sizes to a minimum of 15 and a maximum of 500. Pathways were considered significant based on NES and adjusted *p*-values.

Functional enrichment analysis was performed on a subset of DEGs, defined by a significance threshold of *p <* 0.05 and |log_2_FC| > 0.5. GO enrichment for these DEGs was analyzed using the clusterProfiler package (v.4.10.1). Statistical significance was determined using the Benjamini–Hochberg correction, with a threshold for significant enrichment set at padj ≤ 0.05.

### RT-qPCR

Following reverse transcription of purified RNA (MR05201 Reverse Transcription Kit, Monad Biotech, China), qPCR assays were conducted with the Selleck B21202 2 × SYBR Green qPCR Master Mix on a QuantStudio™ 6 Pro system. All reactions were performed in triplicate, with expression levels normalized to endogenous controls (Gapdh) and analysed via the 2^-ΔΔCt^ method.

### Magnetic resonance imaging (MRI)

MRI was performed on an 11.7 T preclinical MRI system (Bruker BioSpec, Billerica, MA, 117/16) with a Tx/Rx quadrature coil. Higher-resolution T2 weighted image (T2WI) was acquired using a TurboRARE sequence with parameters as follows: repetition time (TR)/echo time (TE) = 550/20.5 msec, slice thickness = 0.5 mm, 8 slices, flip angle = 90, field of view (FOV) = 20 × 25 mm^2^, matrix = 160 × 200, rare factor = 8, average = 30, acquisition time = 6 min 52 s. The extent of lesion heterogeneity was quantified using ImageJ software. Specifically, the width of the hypointense (black) area at the injury site was measured, and the maximum width across all axial sections was recorded.

### Immunofluorescence staining

Paraffin-embedded spinal cord sections were deparaffinized in xylene and rehydrated through a graded ethanol series. Antigen retrieval was performed by submerging slides in EDTA alkaline retrieval buffer (pH 9.0) and heating via microwave for 10 min, followed by natural cooling to room temperature. To quench endogenous peroxidase activity, sections were incubated in 3% hydrogen peroxide for 5 min. After blocking nonspecific binding with 3% bovine serum albumin (BSA) for 1 h at room temperature, sections were incubated with primary antibodies overnight at 4 °C. The primary antibodies used were: COL3A1 (Proteintech), GFAP (Abcam), iNOS (Proteintech), CD206 (Cell Signaling). Following equilibration to room temperature and washing with PBS, sections were incubated with the corresponding secondary antibodies for 1 h in the dark. Fluorescence signals were developed by incubation with a fluorescent reaction solution for 10 min. Nuclei were counterstained with DAPI for 10 min. Slides were washed and mounted using an anti-fade mounting medium, and images were acquired using a multi-channel fluorescence scanner.

For quantitative analysis, all images were acquired using standardized magnification and contrast settings. ImageJ software was employed for region of interest (ROI) selection and morphometric quantification. The fibrotic scar area and the GFAP-deficient zone were determined by manually delineating the boundaries between fluorescence-positive and negative regions surrounding the injury site. Additionally, the mean fluorescence intensity was quantified by defining an ROI centered at the lesion epicenter, extending 1.5 mm rostrally and caudally, to evaluate the average protein expression within this localized injury domain.

### Transmission electron microscopy (TEM)

Fresh spinal cord tissues within the lesion epicenter were rapidly dissected and trimmed into 1 mm^3^ blocks within 3 min to minimize mechanical artifacts, then immediately immersed in electron microscopy fixative at 4 °C for preservation. Following three washes in 0.1 M phosphate buffer (PB, pH 7.4), samples were post-fixed in 1% osmium tetroxide prepared in 0.1 M PB for 2 h at room temperature in the dark. Tissues were then dehydrated through a graded ethanol series (30 to 100%) and pure acetone. Infiltration was performed using a mixture of acetone and EMbed 812 resin (1:1 for 2–4 h, then 1:2 overnight at 37 °C), followed by pure resin infiltration for 5–8 h. Samples were embedded in fresh resin and polymerized at 60 °C for 48 h. Ultrathin sections (70 nm) were cut using an ultramicrotome and collected on 200-mesh Formvar-coated copper grids. Sections were double-stained with 2% uranyl acetate in ethanol for 10 min and lead citrate for 10 min, then examined using a transmission electron microscope.

### Basso mouse scale (BMS)

Mice were placed individually in a circular open-field arena with a non-slippery surface and allowed to move freely for 4 min. Locomotor performance was evaluated by two independent observers who were blinded to the experimental groups to ensure unbiased scoring. Assessments were conducted on days 1, 3, 5, 10, 15, 20, 25, and 30 post-injury (dpi). The BMS scores range from 0 (complete hindlimb paralysis) to 9 (normal locomotion), based on the evaluation of joint movements, stepping frequency, coordination, and trunk stability.

### Motor evoked potential (MEP) assessment

Mice were anesthetized with 2,2,2-tribromoethanol (Avertin, 250 mg/kg, i.p.) and positioned on an insulating platform with the head stabilized and limbs naturally extended. To elicit MEPs, the stimulating anode was placed subcutaneously at the mandible, while the cathode was positioned at the cranial level. Recording electrodes were inserted into the target hindlimb muscle groups. Electrophysiological signals were captured using the Medusa system with pre-optimized filtering parameters. Following the establishment of a stable baseline, stimulation (pulse width: 0.2 ms; intensity: 20 V) was delivered via a stimulator and an isolator. MEPs were identified as biphasic or multiphasic waveforms appearing post-stimulation, with amplitudes at least 2–3 times the baseline noise floor. Data extraction and processing were performed using NeuroExplorer.

### Hindlimb grip strength assessment

Hindlimb grip strength was quantified using a digital grip strength meter (Shanghai Xinruan, China). To minimize environmental stressors, all behavioral tests were conducted in a dedicated room with strictly controlled temperature, humidity, and lighting. The apparatus was calibrated and zeroed before each testing session. Mice were grasped by the base of the tail and positioned such that only the hindlimbs grasped the metal grid. Once a stable grip was established, the mouse was gently and steadily pulled away from the sensor until the grip was released. The peak force (N) exerted by the hindlimbs was recorded from the digital display. This procedure was repeated three times for each mouse, and the values were averaged to determine the mean hindlimb grip strength.

### Hot plate test

Thermal sensitivity was evaluated using a hot plate analgesia meter (SansBio, China). Prior to testing, mice were habituated to a quiet testing environment (22–25 °C) in transparent plexiglass cages to minimize stress-induced confounding factors. To specifically assess hindlimb nociception, the thermal plate was maintained at a constant 55 °C. Mice were secured in a specialized restrainer that immobilized the forelimbs and trunk, ensuring that only the hindlimbs were in contact with the heated surface. The response latency was recorded from the moment of placement until the manifestation of characteristic nociceptive behaviors, defined as toe spreading, hindlimb struggling, or vigorous shaking. Each animal underwent three trials with a 5 min inter-trial interval to ensure recovery and prevent thermal tissue injury. For subsequent analysis, the response speed was calculated as the reciprocal of the reaction time (1/latency).

### Mechanical hypersensitivity assessment

The 50% paw withdrawal threshold (PWT) was determined using a series of calibrated von Frey filaments (North Coast, USA) following the classical “up-down” method. Prior to testing, mice were habituated to the experimental room for 30–60 min. For the assessment, animals were placed in individual transparent plexiglass chambers on an elevated wire mesh platform and allowed to acclimate for an additional 15–30 min until completely calm. The test commenced with the 1.4 g filament, applied to the mid-plantar surface of the right hindlimb (avoiding the footpads) for 5 s with sufficient force to cause slight buckling (S-shape). Given the impaired motor capacity of SCI mice, a positive response was strictly defined as the manifestation of toe spreading, vigorous hindlimb shaking, or intense forelimb struggling; the absence of these behaviors was recorded as a negative response. If no response occurred, the next higher filament was applied; conversely, a positive response triggered the use of the next lower filament. Following the first reversal (a change from positive to negative or vice versa), four additional consecutive measurements were performed to complete the response sequence. A minimum inter-trial interval of 1 min was maintained to prevent sensitization. The 50% PWT was calculated using the formula: 50% PWT (g) = 10^(Xf + κδ-4)^, where parameters were derived using the standard Dixon lookup table.

## Results

### Label-free SHG imaging captures spinal microglial modulation

In our previous work, we established a method to detect microglial motility in the mouse cerebral cortex based on label-free second-harmonic generation (SHG) imaging (Sun, 2019; Sun et al., 2022). Specifically, during the establishment of a mouse cortical thrombosis model, we discovered a rapid increase in SHG signals 10 min after thrombus formation, and confirmed through a series of experiments that these signals corresponded to microglial activity (Sun et al., 2022). This phenomenon closely mirrors the rapid microglial recruitment observed by Stowell et al. in *in vivo* imaging studies (Stowell et al., 2019), and refelects the fact that SHG signals originate from ordered cellular structures such as microtubules and membranes. Based on these studies, we developed a long-term in vivo SHG imaging window at the thoracic level (T10) of the spinal cord to monitor the dynamic changes of spinal microglia (Supplementary Figure S1).

Regarding the modulation of these cells, research by the Tsai lab has shown that 40 Hz audio-visual stimulation induces gamma oscillations and activates microglia in the brain to ameliorate Alzheimer’s pathology (Iaccarino et al., 2016; Martorell et al., 2019), with recent studies confirming efficacy in monkeys (Wang et al., 2026). Following the same logic, in the spinal cord, we sought to apply 40 Hz stimulation to activate microglia. However, given that the spinal cord necessitates more direct stimulation than sensory cortices, we first employed microcurrent stimulation (MCS). To deliver this stimulation safely, we implanted carbon nanotube (CNT) fiber electrodes custom-fabricated by Peng’s group, which are exceptionally soft and exhibit superior tissue compatibility, allowing for stable implantation without inducing chronic inflammation (Wang et al., 2020; Wang et al., 2024).

**Figure 1:**
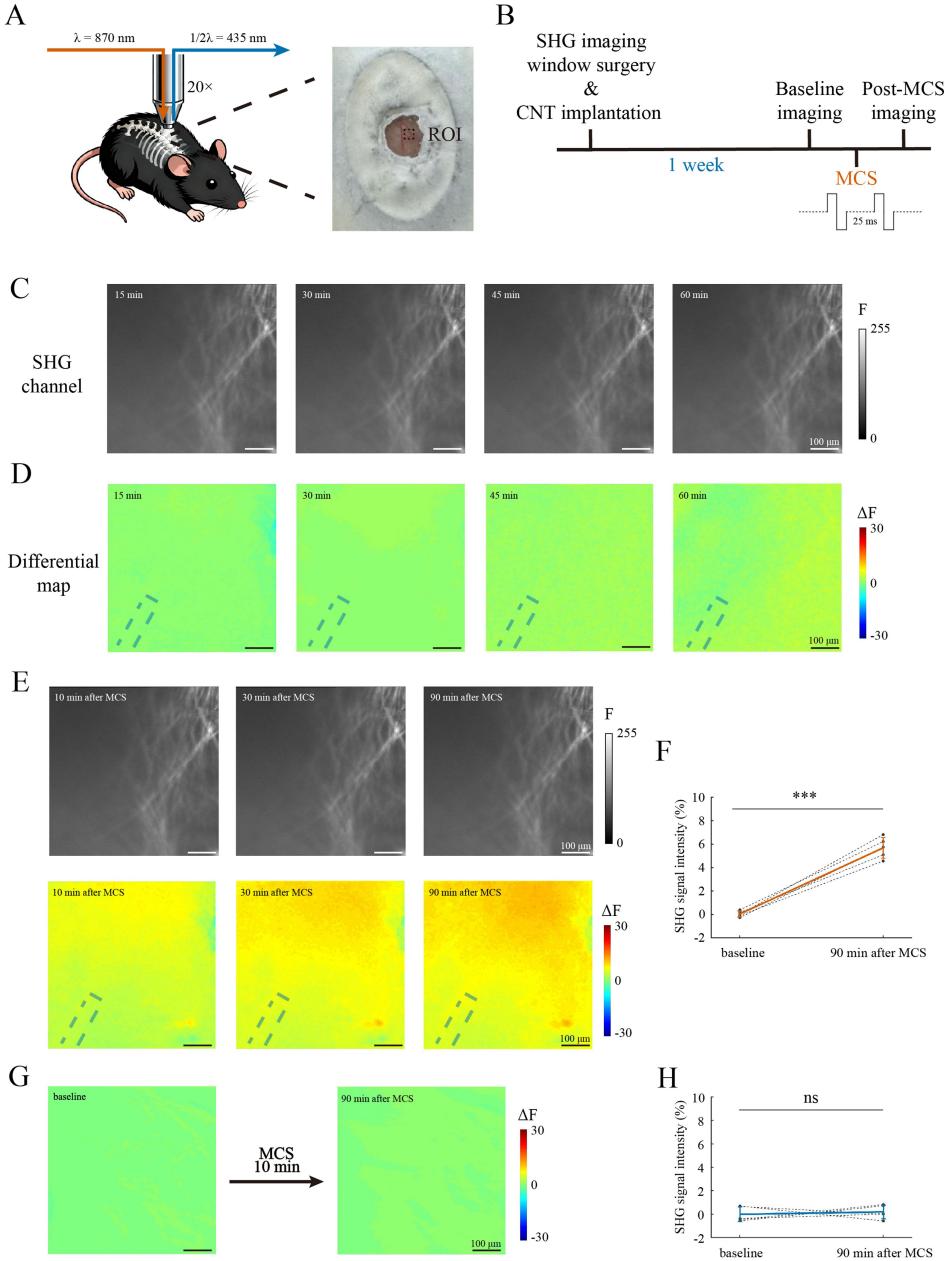
Label-free imaging captures spinal microglia related SHG signal induced by 40 Hz MCS. **(A)** Schematic illustration of the in vivo spinal SHG imaging setup and imaging window. **(B)** Experimental timeline and MCS mode schematic diagram. **(C,D)** SHG signal stability under baseline conditions. Representative raw SHG images **(C)** and corresponding differential heatmaps **(D)** acquired over 60 min without stimulation. The blue dashed boxes indicate the position of the CNT electrode. **(E)** Time-lapse raw SHG images (top row) and corresponding differential heatmaps (bottom row) following 40 Hz MCS. **(F)** Quantification of SHG signal intensity trajectories following 40 Hz MCS. The percentage change in SHG signal (ΔF/F_0_) is calculated as (F_t_ - F_0_)/F_0_, where F_t_ represents the mean whole-field intensity at the indicated time points (60 min of non-intervention, labeled ‘baseline’; and 90 min post-MCS) and F_0_ denotes the initial mean intensity. The solid line indicates the mean percentage change, while dashed lines represent individual trajectories (baseline 0.0366% ± 0.2497% to MCS 5.687% ± 0.8899%, *n =* 5, paired *t*-test). **(G,H)** SHG signal dynamics pre- and post-MCS following PLX3397-mediated microglial depletion. Representative heatmaps **(G)** and quantification **(H)** show negligible signal variation after 40 Hz MCS in microglia-depleted mice (baseline 0.0761% ± 0.6303% to MCS 0.2867% ± 0.5765%, *n =* 5, paired *t*-test). Data are presented as mean ± SEM, *p < 0.05, **p < 0.01, ***p < 0.001 (and ns, *p >* 0.05 where applicable).

As illustrated in the experimental timeline (Figure 1B), since laminectomy and electrode implantation inevitably cause tissue injury, we allowed a recovery period of approximately 1 week to ensure window stabilization before initiating imaging. Imaging was performed using a femtosecond laser tuned to 870 nm, with SHG signals captured at exactly half wavelength of 435 nm through a 20 × water-immersion objective (Figure 1A). It is important to note that unlike fluorescence imaging, SHG imaging cannot directly observe cellular morphology and cell numbers (Figures 1C,E top row). However, SHG excels at capturing global variations within a region of interest (ROI), and by utilizing differential analysis, we can detect minute intensity shifts at the percentage level, which is common in functional imaging techniques, such as instrinsic signal optical imaging and fMRI (Blasdel, 1992; Menon et al., 1997). In this study, we performed recordings at a depth of 0–300 μm. To ensure the consistency of ROI, we utilized the arachnoid reticular fiber structure (0–50 μm depth) for positioning (Figures 1C,E top row). We visualized SHG signal changes by performing differential analysis—subtracting the signal intensity at the initial time point from that at subsequent time points. Before applying stimulation, we validated the stability of the imaging system. Mice were imaged for one hour without intervention, and the differential results demonstrated that the SHG intensity remained stable (Figure 1D).

Subsequently, we administered 40 Hz MCS (40 μA intensity, charge-balanced biphasic pulses; Figure 1B) for 10 min via implanted CNT fiber electrodes. While the raw SHG images still appeared structurally unchanged (Figure 1E, top row), the differential heatmaps captured a rapid and robust signal upregulation across the field of view (Figure 1E bottom). For quantitative analysis, the magnitude of SHG signal was expressed as the percentage change (ΔF/F_0_), calculated as (F_t_ - F_0_)/F_0_, where F_t_ represents the mean whole-field intensity at the indicated time points and F_0_ denotes the initial intensity upon mounting (Figure 1F).

To determine if this signal enhancement was specifically related to microglial dynamics, we pharmacologically depleted microglia by continuously feeding mice a diet containing the CSF1R inhibitor PLX3397, a widely established agent for microglial depletion in the CNS (Elmore et al., 2014), for one month. In microglia-depleted mice, the same 40 Hz MCS protocol failed to elicit any significant change in SHG intensity (Figures 1G,H). Collectively, these data suggest that 40 Hz MCS effectively recruits spinal microglia, a process that can be captured in real-time by our label-free SHG imaging modality.

### 40 Hz rTSMS frequency-specifically induces spinal microglial modulation

While MCS has been proven effective in modulating microglia, the requisite electrode implantation inevitably causes localized tissue damage. To identify a non-invasive intervention with high clinical translational potential, we investigated the influence of repetitive transspinal magnetic stimulation (rTSMS) on microglial behavior. Leveraging the principle of electromagnetic induction, rTSMS generates intraspinal currents comparable to MCS while offering the critical advantage of being non-invasive.

**Figure 2:**
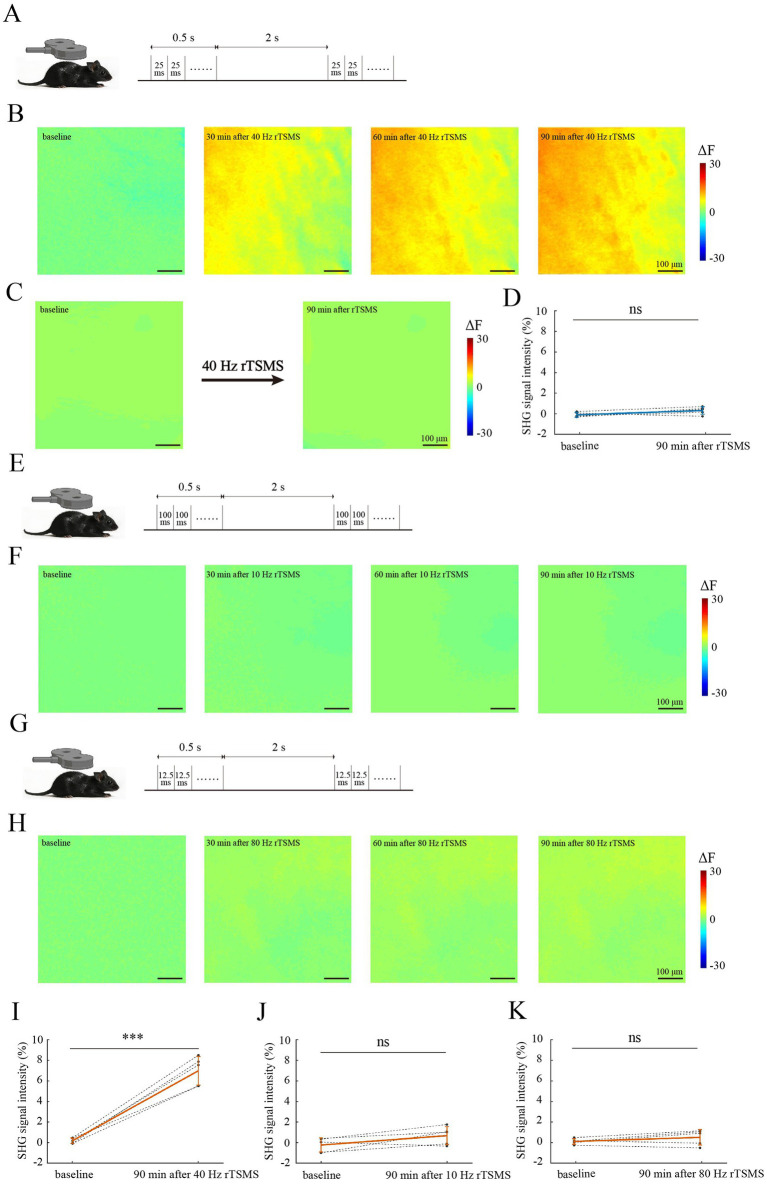
Frequency-specific modulation of spinal microglia induced by non-invasive rTSMS. **(A)** 40 Hz rTSMS mode schematic diagram. **(B,I)** SHG signal changes following 40 Hz rTSMS. Representative time-lapse differential heatmaps **(B)** and corresponding quantification **(I)** pre- and post-rTSMS (baseline 0.1991% ± 0.2607% to rTSMS 6.995% ± 1.394%, *n =* 5, paired *t*-test). **(C,D)** SHG signal dynamics pre- and post-rTSMS following PLX3397-mediated microglial depletion. Representative heatmaps **(C)** and quantification **(D)** show negligible signal variation after 40 Hz rTSMS in microglia-depleted mice (baseline −0.1098% ± 0.2340% to rTSMS 0.3027% ± 0.3695%, *n =* 5, paired *t*-test). **(E,G)** 10 Hz **(E)** and 80 Hz **(G)** rTSMS mode schematic diagram. **(F,H,J,K)** Absence of SHG signal changes at non-resonant frequencies. Neither 10 Hz **(F,J)** nor 80 Hz **(H,K)** stimulation induces significant alterations in SHG signal intensity (baseline 0.1950% ± 0.6859% to 10 Hz rTSMS 0.7267% ± 0.8777%, baseline 0.0462% ± 0.2715% to 80 Hz rTSMS 0.4408% ± 0.7445%, *n =* 5 per group, paired *t*-test). In all quantification plots **(D,I,J,K)**, the solid line represents the mean percentage change (ΔF/F_0_) amoung mice, and dashed lines indicate individual trajectories. Data are presented as mean ± SEM, *p < 0.05, **p < 0.01, ***p < 0.001 (and ns, *p >* 0.05 where applicable).

Employing a figure-eight coil positioned over the T10 segment, we applied rTSMS at 30% maximum stimulator output (Figure 2A). While the baseline spinal SHG signal remained stable, the application of 40 Hz rTSMS (200 cycles, 0.5 s on/2 s off; Figure 2A) induced a progressive and significant enhancement of SHG intensity (Figures 2B,I). Consistent with our MCS findings, the rTSMS-induced SHG elevation was abolished in microglia-depleted mice (Figures 2C,D), confirming that this response is similarly related to microglial recruitment.

We subsequently evaluated the frequency specificity of this recruitment effect. Maintaining a similar stimulation pattern, we tested 10 Hz (Figure 2E) and 80 Hz stimulation (Figure 2G). In the rTSMS literature, various frequencies have been attempted (Chalfouh et al., 2020; Zhai et al., 2024; Song et al., 2025; Wu et al., 2026), but due to the limited number of studies, no single frequency has emerged as the most commonly used standard. In the broader rTMS field, 10 Hz is widely reported to modulate microglia and suppress neuroinflammation (Luo J. et al., 2022; Hong et al., 2022), albeit with some contradictory findings (Eichler et al., 2023). Thus, we chose 10 Hz both to reassess its actual function and to serve as a low-frequency comparator. The 80 Hz frequency was selected as a high-frequency control to test whether higher frequencies produce greater effects and to verify that 40 Hz is uniquely beneficial rather than following a monotonic “higher-is-better” trend. Continuous SHG monitoring revealed that neither 10 Hz nor 80 Hz rTSMS stimulation induced significant changes in the intraspinal SHG signal (Figures 2F,H,J,K). These results establish 40 Hz rTSMS as a frequency-specific non-invasive approach for the targeted recruitment of microglia.

### 40 Hz rTSMS remodels the spinal cord transcriptomic landscape: suppressing fibrotic inflammation and rescuing neuronal function

Having established via SHG imaging that 40 Hz serves as a ‘resonant frequency’ driving microglial motility, a pivotal question arises: does it translate into beneficial therapeutic effects within a pathological context? Given the complex pathology of SCI, a mere increase in microglial abundance does not guarantee repair; rather, it is the functional phenotype—specifically the balance between pro-inflammatory M1 and anti-inflammatory M2 states—that ultimately dictates tissue fate.

To determine whether the microglial recruitment elicited by 40 Hz rTSMS translates into pro-regenerative molecular remodeling, we utilized a murine model of spinal cord crush injury resulting in complete loss of hindlimb sensorimotor function (Supplementary Figure S2). We initiated a 2-week daily 40 Hz rTSMS during the critical window of secondary injury (48 h post-injury) (Yang et al., 2005; Donnelly and Popovich, 2008) and performed high-throughput RNA-seq on spinal cord tissues from the injury epicenter and penumbra after 2-week intervention.

**Figure 3:**
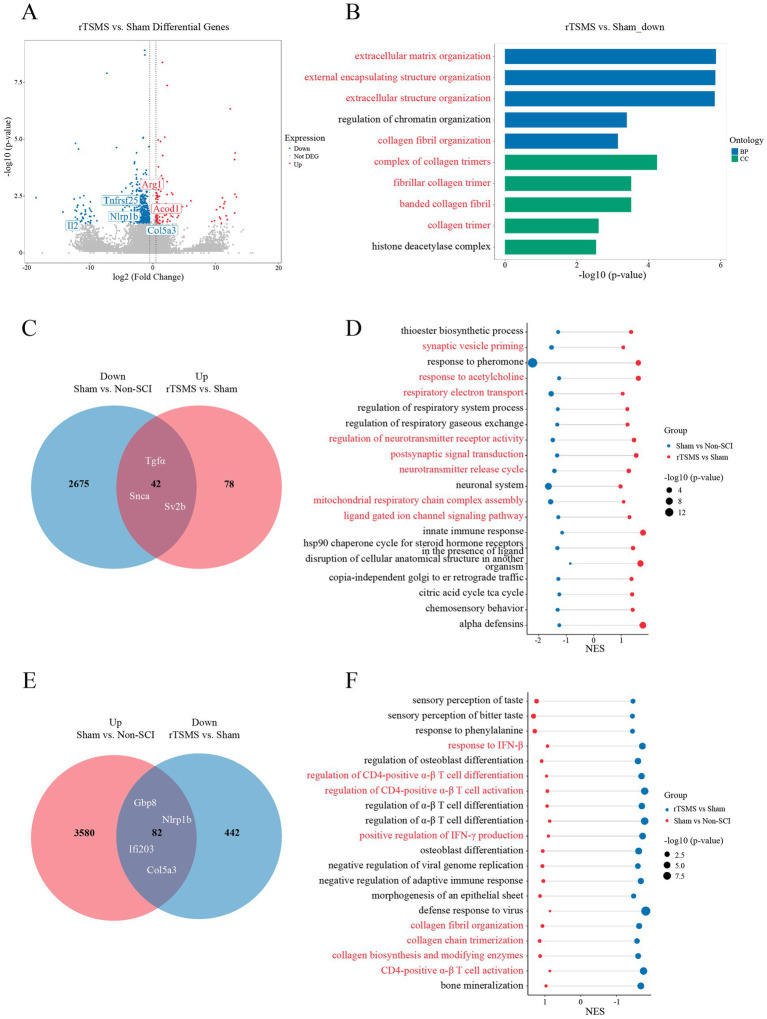
40 Hz rTSMS reprograms the transcriptomic landscape to suppress fibrotic inflammation and rescue neuronal function. **(A)** Volcano plot showing differentially expressed genes (DEGs) in the spinal cord between the 40 Hz rTSMS and Sham groups. Red and blue dots denote significantly upregulated and downregulated genes, respectively. **(B)** GO enrichment analysis of genes significantly downregulated by 40 Hz rTSMS. The top 5 most significantly downregulated pathways within the BP and CC categories are presented. Pathways of particular interest in this study are marked in red. **(C,E)** Venn diagrams illustrating the intersection of genes whose SCI-induced pathological alterations were reversed by 40 Hz treatment. Forty-two genes downregulated by SCI were recovered by treatment **(C)**, while 82 genes upregulated by SCI were suppressed **(E)**. **(D,F)** GSEA identifying functional pathways modulated by 40 Hz rTSMS. Panels display the top 20 pathways with the largest changes in NES in the 40 Hz group compared to the Sham group. NES represents the degree and direction of gene set enrichment, with positive values indicating upregulation and negative values indicating downregulation relative to the comparative group. Statistical significance is expressed as -log10 (*p*-value). Pathways of particular interest in this study are marked in red.

Differential expression analysis revealed a profound reprogramming of the injury microenvironment by 40 Hz rTSMS (Figure 3A). The treatment significantly upregulated regulatory genes associated with anti-inflammation and tissue repair (e.g., *Arg1* and *Acod1*; Figure 3A) (Kigerl et al., 2009; Qian Z. et al., 2024; Qian F. et al., 2024), while concurrently downregulating pro-inflammatory genes (e.g., *Il2*, *Nlrp1b*, *Tnfsf25*; Figure 3A) (Jones et al., 2020; Chui et al., 2019; Kaushal et al., 2015; Valatas et al., 2019) and key collagen fibrillogenesis genes (e.g., *Col5a3*; Figure 3A). The robustness of key differential expression hits was confirmed by RT-qPCR on independent biological replicates (Supplementary Figure S3). Of particular note, *Arg1* is a canonical marker of M2 microglia that plays a pivotal role in synapse maturation and dendritic spine pruning during early development (Miron et al., 2013); consistent with this, Li et al. reported high expression of *Arg1* in microglia mediating scar-free spinal cord repair in neonatal mice (Li et al., 2020). Furthermore, the downregulation of *Col5a3* is critical given its indispensable role in the initiation of collagen fibril assembly (Wenstrup et al., 2004), suggesting that the treatment may impede fibrotic scar formation at its inception (Figure 3A).

Gene Ontology (GO) enrichment analysis further corroborated this anti-fibrotic efficacy. Among the top 5 most significantly downregulated Biological Processes (BP) and Cellular Components (CC), pathways related to extracellular matrix (ECM) organization and collagen fibril organization predominated (Figure 3B). As both processes are integral to scarring post-SCI, these data indicate that 40 Hz rTSMS substantially suppresses pathological scar formation. In parallel, among the top 15 upregulated pathways, they were primarily enriched in the ones governing axons, synaptic structures, and vesicles, highlighting a potential for neural network remodeling (Supplementary Figure S4).

To systematically assess whether 40 Hz rTSMS reverses SCI-induced pathological transcriptomic alterations, we performed an intersectional analysis of data from the rTSMS treated SCI, Non-SCI, and Sham (untreated SCI). We identified 42 genes that were downregulated following SCI but significantly recovered (upregulated) upon 40 Hz intervention (Figure 3C), including the neuroprotective factor *Tgfα* (Dai et al., 2020) and genes essential for synaptic stability and function, such as *Snca* and *Sv2b* (Swaminathan et al., 2019; Paulussen et al., 2023). This indicates that rTSMS rescues neural functions compromised by injury. To further substantiate this “functional rescue” effect, we performed Gene Set Enrichment Analysis (GSEA), specifically focusing on the top 20 pathways exhibiting the largest differences in Normalized Enrichment Score (NES). Since the NES quantifies both the magnitude and direction of gene set enrichment, a large divergence in this score indicates a dramatic shift in gene expression patterns between conditions. These top 20 pathways represent the most profound transcriptional reversal: 40 Hz rTSMS not only reversed the downregulation of synaptic vesicle and neurotransmitter receptor activities but also significantly restored mitochondrial respiratory chain function, thereby mitigating the energy metabolic collapse characteristic of SCI (Figure 3D). Recent reviews have similarly underscored the critical significance of mitochondrial functional recovery in SCI repair (Slater et al., 2022).

On the other hand, we identified 82 genes that were aberrantly upregulated post-SCI but significantly suppressed following treatment (Figure 3E). These genes predominantly encode pro-inflammatory mediators (e.g., *Gbp8*, *Ifi203*) (Man et al., 2017) and fibrotic components. By prioritizing the top 20 pathways exhibiting the largest differences in NES, GSEA analyses consistently demonstrated that 40 Hz rTSMS systemically inhibited SCI-induced IFN-*β*/*γ* inflammatory signaling pathways and CD4-positive T cell infiltration, while synergistically downregulating collagen biosynthetic pathways (Figure 3F). Collectively, these transcriptomic data suggest that 40 Hz rTSMS promotes spinal cord repair via a dual mechanism: specifically abrogating the cytokine storm and fibrotic scar formation, while effectively rescuing compromised neuronal synapses and mitochondrial function.

### 40 Hz rTSMS drives spinal cord repair via frequency-specific transcriptomic remodeling

**Figure 4:**
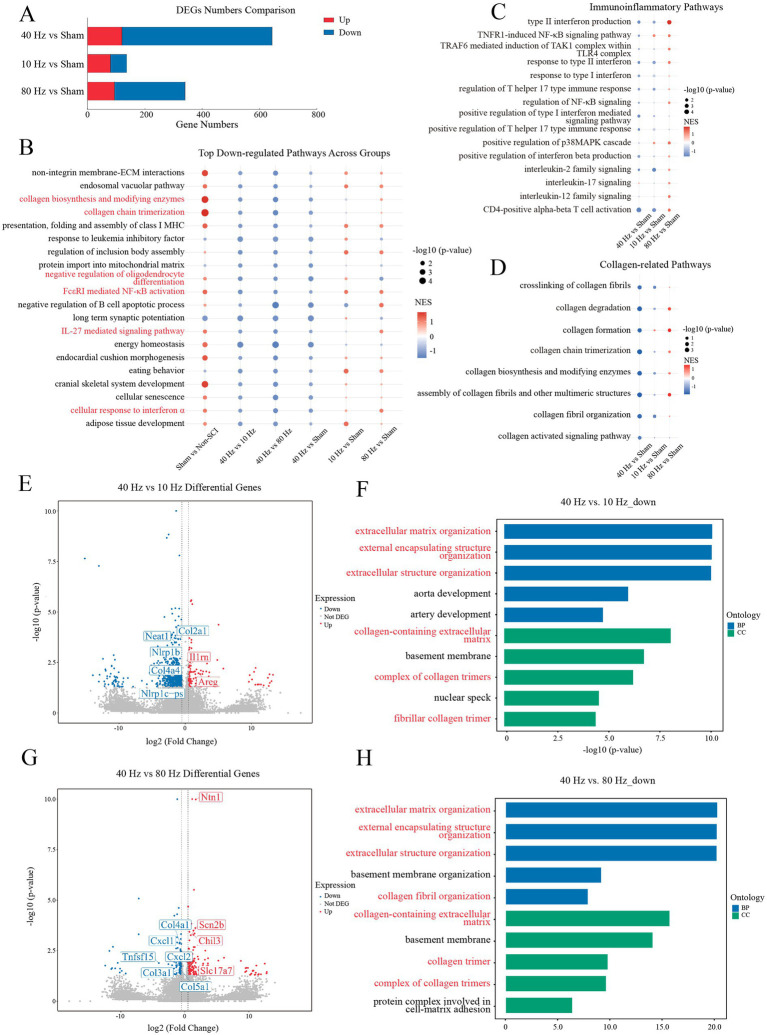
40 Hz rTSMS achieves frequency-specific transcriptomic homeostasis to optimize spinal cord repair. **(A)** Comparison of DEG counts. **(B)** Dot plot of the 20 pathways with largest average NES differences among those downregulated by 40 Hz relative to both 10 Hz and 80 Hz cohorts. Pathways of particular interest in this study are marked in red. **(C,D)** GSEA identifying immuno-inflammatory **(C)** and collagen-related **(D)** pathways modulated by different rTSMS frequencies. **(E,G)** Volcano plots highlighting DEGs in 40 Hz vs. 10 Hz **(E)** and 40 Hz vs. 80 Hz **(G)**. **(F,H)** GO enrichment analysis of BP and CC significantly downregulated by 40 Hz relative to 10 Hz **(F)** and 80 Hz **(H)**. For each comparison, the top 5 most significantly downregulated pathways within the BP and CC categories are presented. Statistical significance is expressed as -log10 (p-value). Pathways of particular interest in this study are marked in red.

To determine whether the therapeutic efficacy of rTSMS is frequency-dependent, we compared the transcriptomic landscapes of the spinal cord following intervention with 40 Hz, 10 Hz, or 80 Hz. We found that 40 Hz stimulation induced a more extensive transcriptomic reprogramming in SCI mice, as reflected by the highest number (120 up, 524 down) of differentially expressed genes (DEGs), compared to 10 Hz (80 up, 57 down) and 80 Hz (95 up, 246 down) interventions (Figure 4A).

Previous studies have noted the anti-inflammatory potential of 10 Hz rTMS (Luo J. et al., 2022; Hong et al., 2022; Yang et al., 2024; Hu et al., 2022; Bai et al., 2023; Han et al., 2023). Our GO analysis confirmed that 10 Hz intervention indeed downregulates several immune-inflammatory pathways (Supplementary Figure S5). However, global GSEA comparisons demonstrated that 40 Hz rTSMS achieved significantly more robust suppression of a randomly selected subset of immune-inflammatory pathways, characterized by more pronounced negative NES (Figure 4C). Direct transcriptomic comparison between 40 Hz and 10 Hz (Figure 4E) showed that 40 Hz uniquely upregulated anti-inflammatory mediators such as *Il1rn* (interleukin-1 receptor antagonist) (Cao et al., 2023) and the tissue-repair factor *Areg* (Zaiss et al., 2015). Conversely, it more effectively suppressed key pro-inflammatory drivers (Figure 4E), including the inflammasome component *Nlrp1b* and the long non-coding RNA *Neat1*, a known promoter of M1 microglial polarization (Chen T. et al., 2024; Chen K. et al., 2024; Pan et al., 2024). This suggests that 40 Hz maximizes the activation of an anti-inflammatory microglial phenotype beyond the capacity of 10 Hz.

Crucially, while 10 Hz showed modest anti-inflammatory efficacy, its impact on remodeling ECM was negligible. In contrast, 40 Hz rTSMS exhibited a unique capacity for fibrosis inhibition. GSEA revealed that pathways related to collagen biosynthesis and collagen chain trimerization were severely attenuated specifically in the 40 Hz group—an effect largely absent in the 10 Hz and 80 Hz cohorts (Figure 4D). This frequency-specific signature implies that 40 Hz not only modulates acute inflammation but also physically obstructs fibrotic scar deposition, thereby preserving a permissive environment for neural regeneration. Direct comparisons between the 40 Hz and 10 Hz groups further confirmed the frequency-specific efficacy of 40 Hz in suppressing fibrosis. 40 Hz stimulation led to a significant downregulation of collagen-encoding genes, such as *Col4a4* (Figure 4E). Moreover, GO enrichment analysis revealed that among the top 5 most significantly downregulated BP and CC, pathways related to ECM organization and collagen fibril organization were predominantly suppressed (Figure 4F).

In stark contrast, high-frequency 80 Hz stimulation contributed little to regenerative pathways and, in some instances, appeared to exacerbate inflammation and fibrosis (Figures 4C,D; Supplementary Figure S6). Unlike the detrimental or neutral effects, 40 Hz treatment uniquely promoted genes associated with neuronal survival and synaptic function. These included the glutamate transporter *Slc17a7* (VGLUT1), the voltage-gated sodium channel *Scn2b*, and the axon guidance cue *Ntn1* (Netrin-1)—the latter of which is known to re-polarize microglia toward an anti-inflammatory M2 state (Yin et al., 2024; Moore et al., 2012) (Figure 4G). Consistent with this, the classic M2 marker *Chil3* was significantly upregulated in the 40 Hz group (Figure 4G). GO analysis corroborated that among the top significantly enriched terms, 40 Hz specifically enriched terms related to ion channels and synaptic structure (Supplementary Figure S7) while suppressing ECM-related pathways (Figure 4H), highlighting its superior potential for neural circuit remodeling.

Finally, a global cross-analysis of the top 20 pathways selected based on the largest average NES differences among those downregulated by 40 Hz relative to both 10 Hz and 80 Hz identified a distinct “molecular fingerprint” unique to 40 Hz (Figure 4B). Detailed examination of these top divergent pathways highlighted the prominence of critical pathological networks, specifically those governing collagen synthesis, NF-κB activation, and the negative regulation of oligodendrocyte differentiation—all of which constitute major barriers to SCI repair. The sustained upregulation of these networks in the Sham group aligns with the severe secondary injury profile typically seen after SCI. Similarly, the 80 Hz group exhibited detrimental activation patterns in these pathways. While the 10 Hz group displayed sporadic improvements, 40 Hz uniformly induced a beneficial downregulation of these pathological networks relative to the Sham baseline, effectively reversing the injury-associated molecular signature (Figure 4B).

### 40 Hz rTSMS ameliorates lesion pathology and modulates microglial polarization

**Figure 5:**
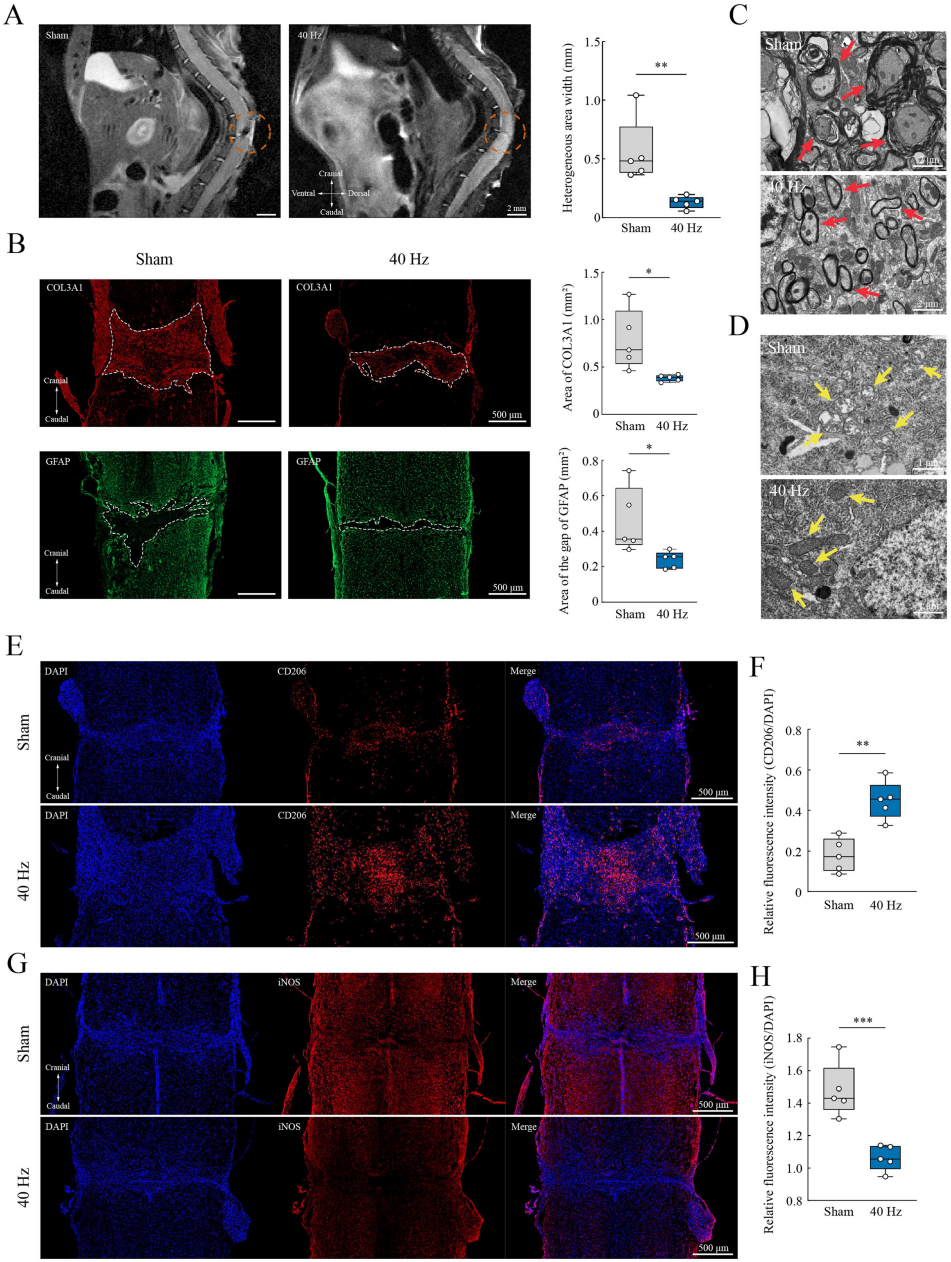
40 Hz rTSMS ameliorates lesion pathology, preserves ultrastructure, and modulates microglial polarization. **(A)** Representative T2-weighted MRI scans of the spinal cord 2 weeks post-injury. The dashed circles indicate the lesion epicenter. Quantification reveals the width of the heterogeneous signal area in each group (Sham 0.56 ± 0.28 mm vs. rTSMS 0.13 ± 0.05 mm, *n =* 5, unpaired *t*-test). **(B)** Immunofluorescence staining for the fibrotic scar marker COL3A1 (red) and the astrocytic marker GFAP (green) at the lesion site (Sham 0.7858 ± 0.3163 mm^2^ vs. rTSMS 0.3828 ± 0.0350 mm^2^ in COL3A1, Sham 0.4578 ± 0.1847 mm^2^ vs. rTSMS 0.2388 ± 0.0474 mm^2^ in GFAP, *n =* 5 per group, unpaired *t*-test). **(C,D)** Representative TEM images of the injury epicenter. Red arrows **(C)** indicate myelinated axons; yellow arrows **(D)** point to mitochondria. **(E,F)** Assessment of M2 microglial polarization. Representative immunofluorescence images **(E)** and relative fluorescence intensity quantification **(F)** of the anti-inflammatory marker CD206 (Sham 0.1792 ± 0.0826 vs. rTSMS 0.4493 ± 0.0940, *n =* 5, unpaired *t*-test). **(G,H)** Assessment of M1 microglial polarization. Representative images **(G)** and relative fluorescence intensity quantification **(H)** of the pro-inflammatory marker iNOS (Sham 1.477 ± 0.1647 vs. rTSMS 1.063 ± 0.0779, *n =* 5, unpaired *t*-test). Data are presented as mean ± SEM, *p < 0.05, **p < 0.01, ***p < 0.001 (and ns, *p >* 0.05 where applicable).

To validate the therapeutic efficacy of 40 Hz rTSMS on spinal structural integrity following SCI, we first assessed gross lesion morphology using 11.7 T magnetic resonance imaging (MRI). T2-weighted imaging at 2 weeks post-injury revealed that 40 Hz treatment significantly reduced the width of the heterogeneous signal area at the epicenter by 76.4% compared to Sham controls (Figure 5A). Corroborating this macroscopic finding, histological analysis via Luxol fast blue (LFB) staining revealed that the gap width of myelin loss was markedly narrower in the rTSMS group (Supplementary Figure S8).

We next examined the formation of the scar barrier, a major impediment to neural regeneration. Following 2-week 40 Hz rTSMS treatment, a significant decrease in the COL3A1-defined fibrotic scar area was observed, along with a reduction in the GFAP-deficient area (Figure 5B). The 40 Hz group exhibited a 51.3% decrease in fibrotic scar area and a 47.8% reduction in the GFAP-deficient lesion size. At the ultrastructural level, transmission electron microscopy (TEM) captured at the lesion epicenter provided further evidence of neuroprotection. The sham group exhibited severe myelin pathology, characterized by uneven sheath thickness, pronounced swelling, and degeneration of the lamellar structure, resulting in a loose and disorganized arrangement (Figure 5C top). In contrast, axons in the 40 Hz group maintained preserved myelin integrity, displaying sheaths of uniform thickness devoid of the significant thickening, degeneration, or lamellar separation observed in controls (Figure 5C bottom). Regarding mitochondrial health, organelles in the Sham group displayed signs of severe structural damage, including swelling, vacuolization, outer membrane rupture, matrix loss, and fragmented or scarce cristae (Figure 5D top). Conversely, the 40 Hz treated tissue contained abundant mitochondria that predominantly exhibited intact outer membranes, well-defined cristae architecture, and overall normal morphology (Figure 5D bottom).

Finally, to link these structural improvements to the inflammatory microenvironment, we characterized microglial phenotypes within the lesion site. Consistent with a shift towards a reparative state, the 40 Hz group showed a significant upregulation of the anti-inflammatory marker CD206 (Figures 5E,F) and a concurrent reduction in the pro-inflammatory marker iNOS (Figures 5G,H). Collectively, these data indicate that 40 Hz rTSMS fosters a pro-restorative environment by mitigating scarring, preserving myelin ultrastructure, and promoting an M2-dominant microglial polarization.

### 40 Hz rTSMS facilitates multi-modal functional restitution after SCI

To determine whether the 40 Hz rTSMS-induced molecular and structural remodeling at the lesion site translated into tangible functional benefits, we performed a systematic assessment of locomotor and sensory recovery post-SCI.

**Figure 6:**
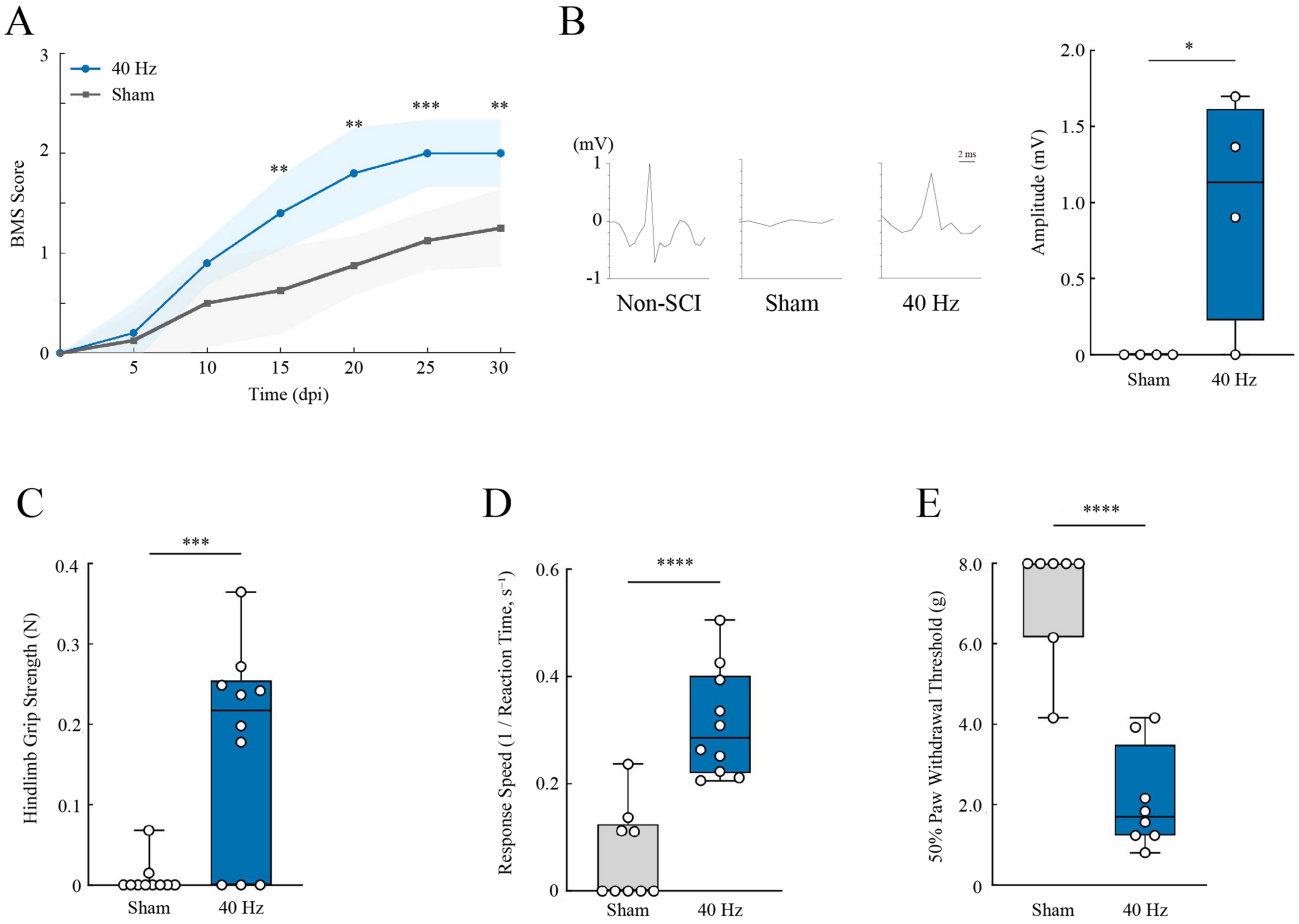
40 Hz rTSMS facilitates multi-modal motor and sensory functional restitution. **(A)** Time course of locomotor recovery assessed using the BMS score over 30 dpi. Shaded areas represent s.d. (*n =* 8 in sham group, *n =* 10 in 40 Hz group, unpaired *t*-test). **(B)** Electrophysiological assessment of corticospinal tract integrity. Representative MEP traces (left) and quantification of peak-to-peak amplitude (right) at 2 weeks post-injury (Sham undetectable vs. rTSMS 0.9915 ± 0.7373 mV, *n =* 4, unpaired *t*-test). **(C)** Quantification of hindlimb grip strength (Sham 0.0083 ± 0.0215 N vs. rTSMS 0.1741 ± 0.1298 N, *n =* 10, unpaired *t*-test). **(D)** Evaluation of thermal nociception via the hot plate test. Response speed was calculated as the reciprocal of the reaction latency (1/reaction time); animals not responding within the 10 s cutoff were assigned a speed of 0 (Sham 0.0664 ± 0.0868 s^−1^ vs. rTSMS 0.3123 ± 0.1015 s^−1^, *n =* 9 in sham group, *n =* 10 in rTSMS group, unpaired *t*-test). **(E)** Assessment of mechanical sensation using von Frey filaments. A cutoff of 8 g was applied; responses exceeding this limit were recorded as 8 g (Sham 7.189 ± 1.501 g vs. rTSMS 2.116 ± 1.261 g, *n =* 7 in sham group, *n =* 8 in rTSMS group, unpaired t-test). Data are presented as mean ± SEM, *p < 0.05, **p < 0.01, ***p < 0.001, ****p < 0.0001 (and ns, *p >* 0.05 where applicable).

The long-term trajectory of locomotor recovery was first quantified using the Basso Mouse Scale (BMS). In this experiment, rTSMS treatment was continued daily for the entire 30-day observation period. Although both experimental groups exhibited initial complete paralysis, mice receiving 40 Hz rTSMS treatment displayed a significantly superior recovery trend compared to the sham-treated cohort starting from 2 weeks post-injury. Specifically, the 40 Hz group progressed to demonstrate extensive ankle movement and dorsal stepping to support locomotion, whereas the untreated group was restricted to only slight ankle movement throughout the 30-day observation window (Figure 6A; Supplementary Figure S9; Supplementary Movies S1, S2). These behavioral improvements were corroborated by neuroelectrophysiological findings. To assess corticospinal tract integrity, we examined motor-evoked potentials (MEPs) by transcranially stimulating the primary motor cortex using electrodes and recording electromyographic signals from the hindlimb muscles. At 2 weeks post-SCI, corticospinal tract conduction was nearly abolished in sham-treated mice; however, 40 Hz rTSMS treatment significantly rescued MEP signals (Figure 6B). Consistent with these findings, rTSMS-treated mice exhibited a striking elevation in active hindlimb grip strength (Figure 6C), providing direct evidence for the substantial reinforcement of spinal motor output.

Beyond motor control, we examined the influence of 40 Hz rTSMS on sensory system reorganization. We evaluated thermal nociception using the hot plate test (constant 55 °C). Response speed was calculated as the reciprocal of the reaction latency (1/reaction time); animals not responding within the strict 10 s cutoff were considered to lack pain perception, and their response speed was recorded as 0. The 40 Hz group showed a significantly faster response speed (Figure 6D), indicating a partial restoration of nociceptive pathways. Furthermore, mechanical sensitivity was assessed via von Frey filaments using the up-down method. A cutoff of 8 g was applied, where responses exceeding this limit were uniformly recorded as 8 g. While sham mice exhibited sensory loss (high thresholds), 40 Hz treatment significantly lowered the 50% paw withdrawal threshold (Figure 6E).

Taken together, these data demonstrate that 40 Hz rTSMS acts as a potent therapeutic intervention that comprehensively facilitates multi-modal motor and sensory restitution following SCI.

## Discussion

The pursuit of functional restoration following SCI has evolved into a diverse therapeutic landscape (Courtine and Sofroniew, 2019). Biological substitution strategies, primarily stem cell transplantation, aim to replenish lost cellular populations (Martin-Lopez et al., 2021; Sugai et al., 2025; Shen et al., 2025). In parallel, structural engineering approaches utilize biomaterial scaffolds to physically bridge the lesion cavity, providing mechanical support to guide axonal regrowth across the gap (Katoh et al., 2019; Chen T. et al., 2024; Chen K. et al., 2024; Zhu et al., 2025). Simultaneously, the frontier of functional reconstruction has been expanded by neuroprosthetic technologies—such as brain-spine interfaces (BSI)—which engineer “digital bridges” to bypass the lesion entirely (Lorach et al., 2023; Shiferaw et al., 2025). Complementing these biological and engineering modalities, we here present a paradigm shift towards non-invasive endogenous repair by establishing 40 Hz rTSMS as a precise immunomodulatory intervention. By specifically recruiting and repolarizing microglia, 40 Hz rTSMS effectively re-engineers the hostile injury ecosystem into an anti-inflammatory and anti-fibrotic milieu, thereby driving the adult spinal cord toward a reparative state through purely endogenous modulation. Crucially, this strategy functions not as a competitor but as a foundational complement to emerging therapeutics, helping to maximize the engraftment of transplanted stem cells, the integration of biomaterial scaffolds, and the structural preservation necessary for neuroprosthetic functional reconstruction.

In TMS research, a longstanding bottleneck is the reliance on empirical trial-and-error. The “infinite parameter space” composed of mode, intensity, frequency, and interval sequences limits the optimization of therapeutic efficacy and clinical translation (Caulfield and Brown, 2022; Jung et al., 2025). Although studies have attempted to explore these parameters (Somaa et al., 2022; Sveva et al., 2025; Jung et al., 2025), they are lack of suitable methods for directly observing the cellular effects of treatment *in vivo* in real time. The label-free SHG imaging technique introduced in this study successfully achieved the visualization of microglial activation within the spinal cord. This technical breakthrough holds dual significance: methodologically, it eliminates the dependence on transgenic animals or exogenous markers, overcoming issues of chemical toxicity or photobleaching and enabling observation in a near-physiological state (Icha et al., 2017); scientifically, it establishes a direct causal link between stimulation parameters—specifically frequency—and a key innate immune cell behavior. Through this imaging window, we were able to discover the rapid microglia related signals specifically driven by 40 Hz. This marks a paradigm shift in neuromodulation research from “black box” trial-and-error based on macroscopic endpoints to “visualized” precision parameter screening guided by cellular dynamics.

A core finding of our work is that the therapeutic efficacy of rTSMS is strictly frequency-dependent. Previous investigations into magnetic stimulation have yielded a fragmented landscape regarding microglial modulation. While protocols such as 10 Hz, 20 Hz and iTBS have demonstrated potential in promoting anti-inflammatory polarization (Luo J. et al., 2022; Hong et al., 2022; Zong et al., 2020; Luo L. et al., 2022; Zhu et al., 2024; Caglayan et al., 2019; Qian Z. et al., 2024; Qian F. et al., 2024; Cao et al., 2022; Yang et al., 2024; Hu et al., 2022; Bai et al., 2023; Han et al., 2023), their effects remain inconsistent, with reports of unchanged morphological dynamics (Eichler et al., 2023) or even exacerbated neuroinflammation (Muri et al., 2020). Crucially, the majority of these insights are derived from cortical stimulation, leaving the optimal parameters for direct rTSMS largely undefined (Chalfouh et al., 2020; Zhai et al., 2024; Song et al., 2025; Wu et al., 2026). This prevailing uncertainty underscores the necessity of our study, which systematically identifies 40 Hz as the precise, evidence-based frequency required to orchestrate spinal microglial modulation and repair. Consistent with some prior studies (Luo J. et al., 2022; Hong et al., 2022; Zhai et al., 2024; Yang et al., 2024; Hu et al., 2022; Bai et al., 2023; Han et al., 2023), our transcriptomic data confirm that 10 Hz stimulation contributes to the suppression of pro-inflammatory responses. However, in vivo SHG imaging revealed a critical mechanistic difference: 10 Hz stimulation failed to induce significant microglia related signals, whereas 40 Hz uniquely drove them. It orchestered with the detailed comparative transcriptomic analysis, showing that 40 Hz induces a stronger and more robust pro-repair program than 10 Hz. Importantly, 40 Hz rTSMS acts as a dual-function switch: it not only recruits microglia and reprograms them into an M2 phenotype (upregulating Arg1, CD206), but more importantly, it simultaneously downregulates fibrosis-related pathways—an effect that was not significant in the 10 Hz group. In contrast, higher-frequency 80 Hz stimulation not only failed to promote repair but paradoxically exacerbated inflammatory responses, indicating that “higher is not better,” but rather highlighting 40 Hz as a unique characteristic. Actually in previous studies: Li et al. demonstrates that specific microglial subpopulations in neonatal mice are essential for preventing fibrotic scar formation and allowing the “scar-free” repair required for axonal regeneration (Li et al., 2020). Extending this potential to the adult CNS, Xu et al. recently identified specific signaling pathways capable of reprogramming microglia to adopt a similar scar-free reparative phenotype in adult mice (Xu X. et al., 2025). By inducing this state in adult mice via 40 Hz rTSMS, we effectively reprogrammed the hostile injury microenvironment into one permissive for regeneration, a fact confirmed by the preserved myelin ultrastructure and restored corticospinal tract conduction (MEP) observed in our results.

The 40 Hz rTSMS strategy proposed in this study provides a novel non-invasive paradigm for the treatment of severe physical spinal cord injury, and greatly extends previous studies in mild and neurodegenerative diseases in the brain, in which 40 Hz stimulation recruits microglia to clear amyloid plaques in Alzheimer’s disease models (Iaccarino et al., 2016; Martorell et al., 2019; Wang et al., 2026). This implies that microglia possess a conserved, systemic sensitivity to 40 Hz oscillations. This concept of modulating intrinsic immune cells to counteract secondary fibrotic pathology may be applicable to a range of CNS disorders that share a common end-stage pathology of chronic inflammation and fibrosis.

While promising, our study has limitations that delineate the path for future research. First, the detailed molecular pathways through which 40 Hz oscillatory signals influence microglia remain to be fully elucidated. Second, while we systematically screened stimulation frequencies, other critical parameters, such as total cycles, inter-train interval, on/off cycle, and stimulus intensity, were held constant in this study. The stimulation pattern we selected was pragmatic, balancing a total session time near the 10-min commonly used in the literature with the technical constraint of coil overheating. Future studies should systematically explore this multidimensional parameter space to define the true therapeutic window for rTSMS. Third, in the present study, we primarily applied rTSMS during the early secondary injury phase (starting 48 h post-SCI). While this approach allowed us to identify 40 Hz as the optimal frequency and to characterize its acute protective effects, chronic spinal cord injury represents a major clinical challenge that we have not yet fully addressed. Therefore, a key direction for our future research is to systematically evaluate the efficacy of 40 Hz rTSMS in chronic-stage SCI models and to explore the underlying mechanisms that may enable it to overcome the pathological barriers of the chronic injury milieu. Finally, the sensorimotor recovery observed in complete crush injury model, though robust, did not amount to a complete cure. However, as emphasized in our preceding discussion, this outcome highlights the strategic value of 40 Hz rTSMS not as a standalone panacea, but as a foundational platform for combinatorial therapies. By endogenously fostering a permissive microenvironment for repair, our protocol unlocks immense potential for synergistic integration with rehabilitation training, pharmacological agents, or bio-engineering strategies to maximize functional restoration. Most importantly, as a none-invasive physical therapy, rTSMS is clinically translational and ready-to-use in multiple CNS injuries, and prominent recovery is especially promising in partial and mild cases.

## Data Availability

The datasets presented in this study can be found in online repositories. The names of the repository/repositories and accession number(s) can be found in the article/Supplementary material.
