# Supplementary material for: Screening the optimal rTSMS frequency to orchestrate immune-fibrotic remodeling for adult spinal cord repair
Source: Front Neurosci. 2026 Jun 16;20:1845752. doi: 10.3389/fnins.2026.1845752 (PMC13315000; doi:10.3389/fnins.2026.1845752)
Supplement: Supplementary file 1 [file Data_Sheet_1.PDF]

Fig. S1

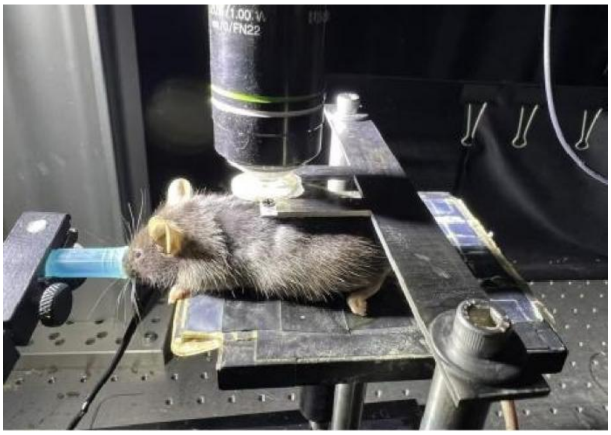

The custom-designed in vivo imaging setup. The anesthetized mouse is immobilized on a stable platform, allowing the 20× water-immersion objective to be positioned directly over the optical window for real-time label-free SHG signal acquisition.

Fig. S2

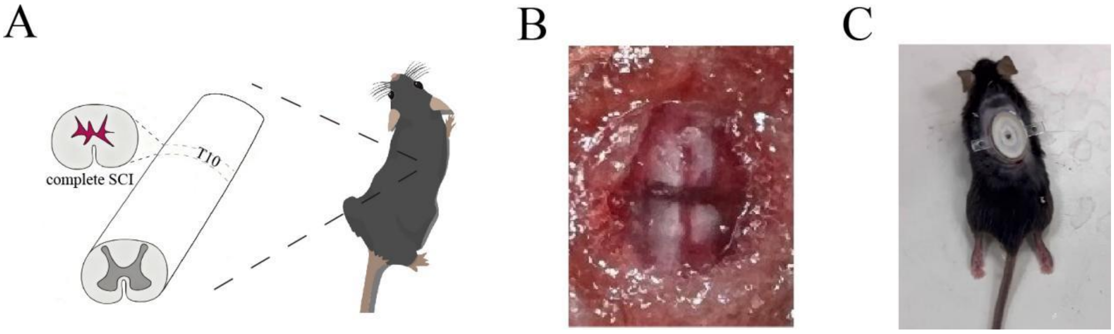

Schematic of the complete spinal cord crush injury model. **A** Diagram depicting the location of the severe crush injury at the thoracic T10 spinal cord segment. **B** Morphology of the injury site immediately post-crush. **C** Loss of hindlimb motor function after SCI. The mouse exhibits flaccid paralysis and dragging of the hindlimbs.

Fig. S3

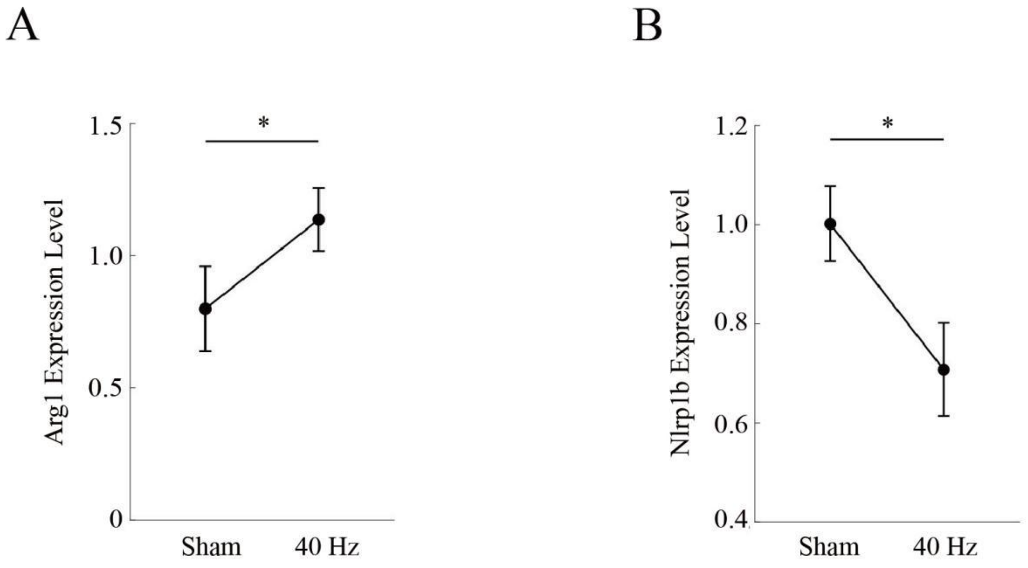

40 Hz stimulation modulates the expression of inflammation-related genes. **A, B** Relative mRNA expression levels of *Arg1* (a) and *Nlrp1b* (b) measured by RT-qPCR in the Sham and 40 Hz groups. mRNA levels were normalized to Gapdh. Data are presented as mean ± SEM, unpaired t-test, n = 3, *$p < 0.05$, **$p < 0.01$, ***$p < 0.001$ (and ns, $p > 0.05$ where applicable).

Fig. S4

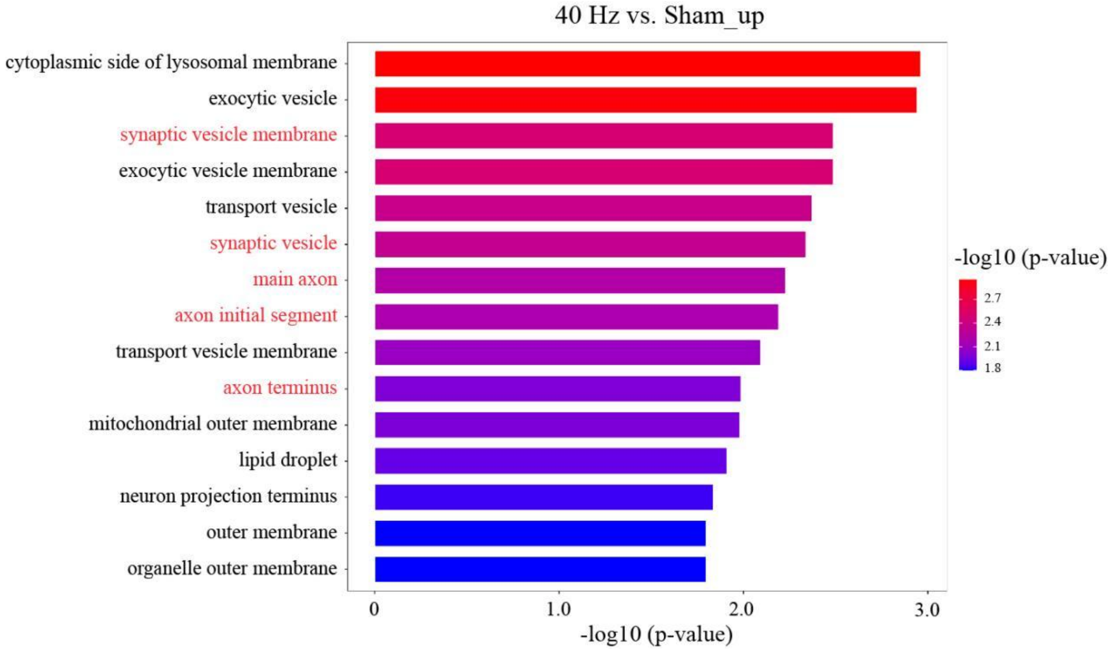

40 Hz rTSMS promotes transcriptomic pathways associated with neural network remodeling. GO enrichment analysis of genes significantly upregulated in the 40 Hz group relative to Sham. The top 15 most significantly upregulated pathways within CC categories are presented. Statistical significance is expressed as -log10 (p-value).

Fig. S5

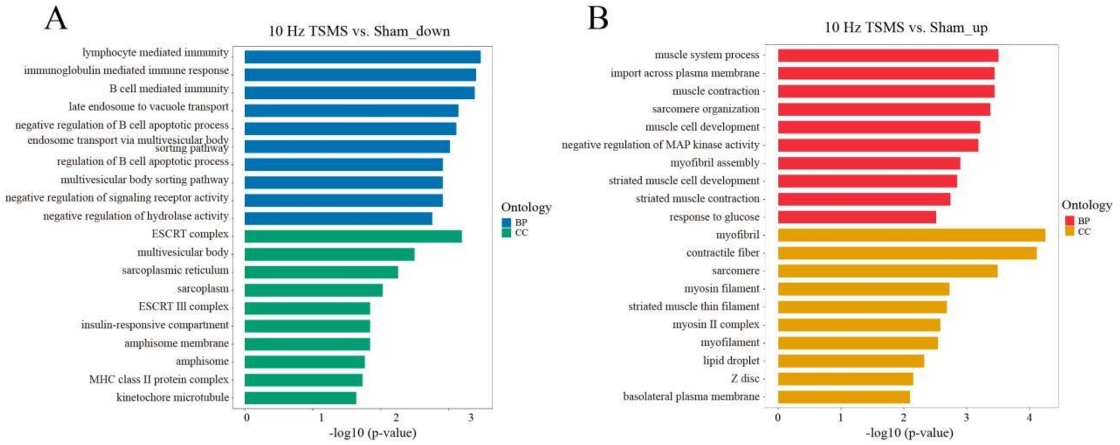

GO enrichment analysis of the spinal cord transcriptomic response to 10 Hz rTSMS. **A, B** GO enrichment analysis of genes significantly downregulated (**A**) or upregulated (**B**) in the 10 Hz rTSMS group compared to the Sham group. For each panel, the top 10 most significantly enriched pathways within the BP and CC categories are displayed. Statistical significance is expressed as -log10 (p-value).

Fig. S6

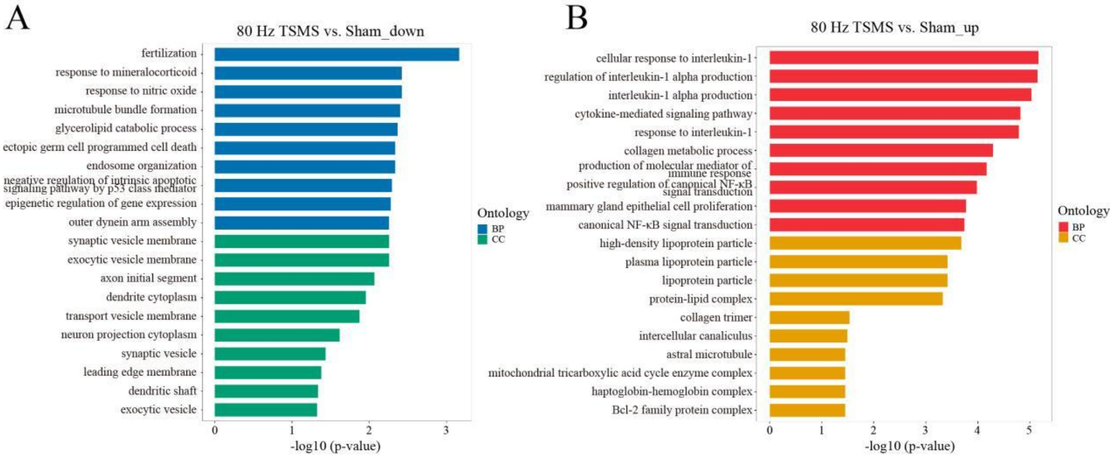

GO enrichment analysis of the spinal cord transcriptomic response to 80 Hz rTSMS. **A, B** GO enrichment analysis of genes significantly downregulated (**A**) or upregulated (**B**) in the 80 Hz rTSMS group compared to the Sham group. For each panel, the top 10 most significantly enriched pathways within the BP and CC categories are displayed. Statistical significance is expressed as -log10 (p-value).

Fig. S7

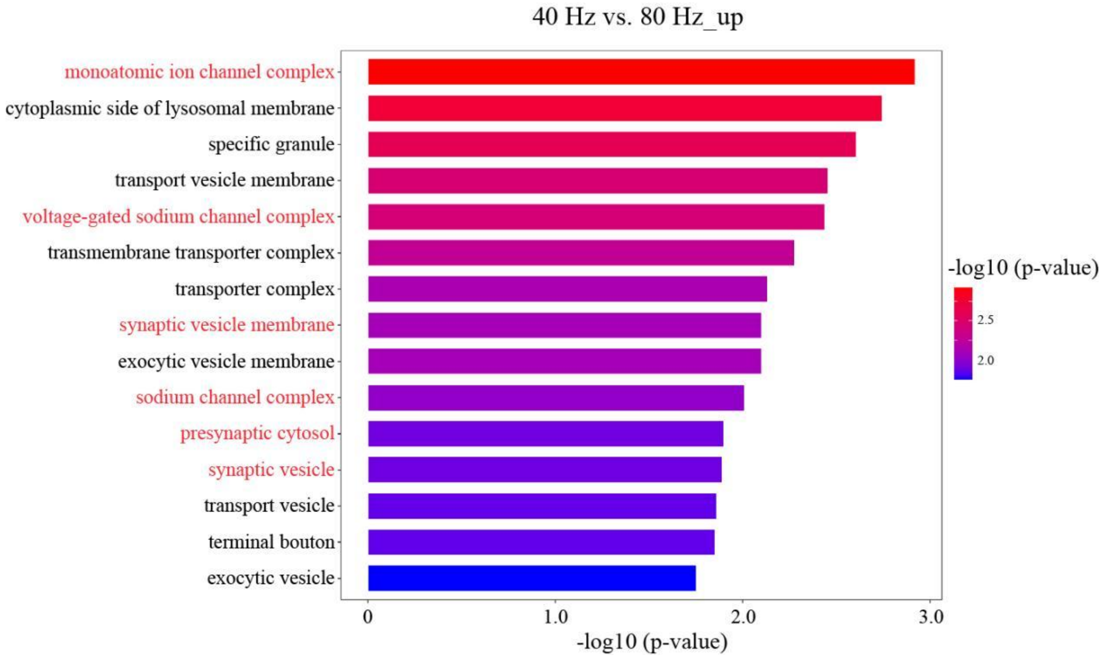

40 Hz rTSMS uniquely promotes neural structural and functional pathways compared to 80 Hz stimulation. GO enrichment analysis of genes significantly upregulated in the 40 Hz group relative to the 80 Hz group. The plot displays the top 15 most significantly upregulated CC pathways, highlighting a distinct enrichment in terms related to ion channel complexes and synaptic structures. Statistical significance is expressed as -log10 (p-value).

Fig. S8

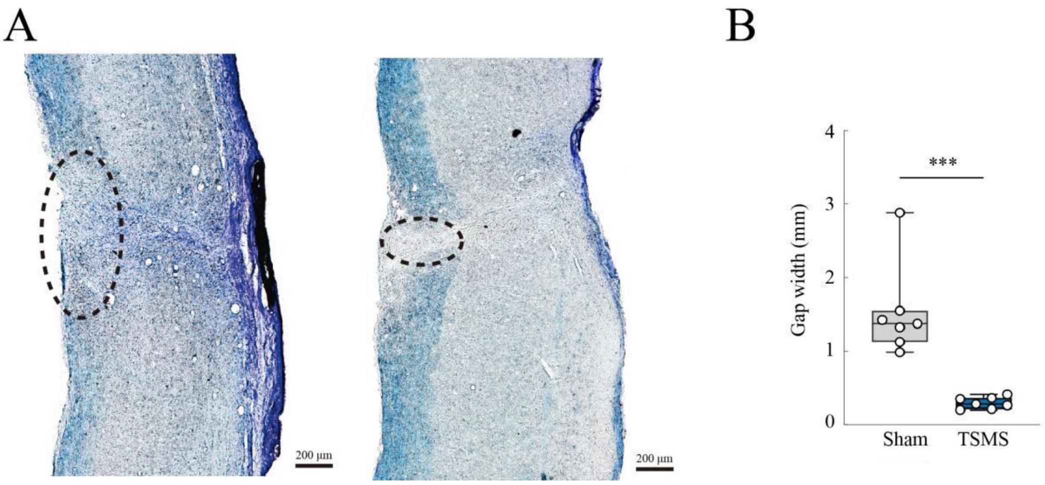

40 Hz rTSMS attenuates myelin loss. **A** Representative LFB-stained longitudinal sections 2 weeks post-injury. Dashed ovals outline the demyelinated lesion gap (loss of blue staining). **B** Quantification of the gap width. Data are presented as mean ± SEM, unpaired t-test, n = 7, *p < 0.05, **p < 0.01, ***p < 0.001 (and ns, p > 0.05 where applicable).

Fig. S9

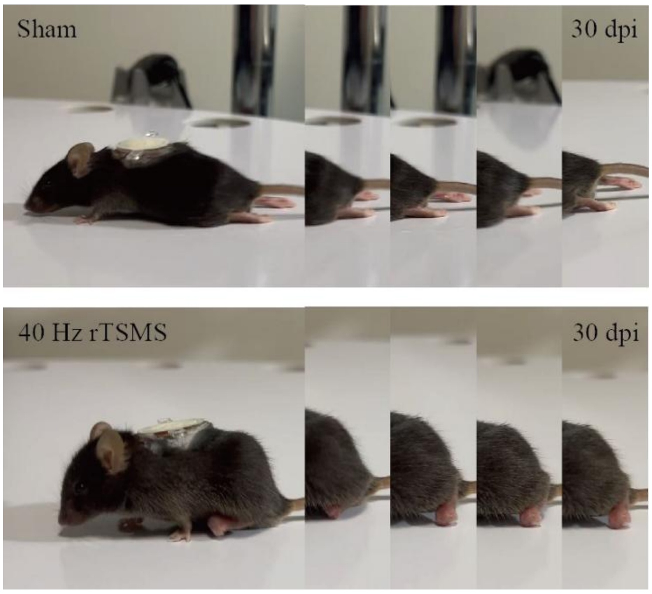

Representative images of hindlimb movement in mice at 30 dpi with (bottom) or without (top) 40 Hz rTSMS.
